# Orthodontic traction of impacted canines: Concepts and clinical application

**DOI:** 10.1590/2177-6709.24.1.074-087.bbo

**Published:** 2019

**Authors:** Ricardo Machado Cruz

**Affiliations:** 1 Private practice (Brasília/DF, Brazil).

**Keywords:** Angle Class II, Deep bite, Canine traction, Impacted canines, Corrective Orthodontics

## Abstract

Orthodontic traction of impacted canines stands as a major challenge for Orthodontics. It is a relatively frequent clinical complaint which management, more often than not, requires a multidisciplinary approach. Surgical exposure of the impacted canine, and the complex orthodontic mechanics applied to align the tooth back into the arch, may frequently lead to complications involving supporting tissues, not to mention the long treatment time and high costs imposed to patients. In face of that, it is worth highlighting the relevance of early diagnosis as to intervene efficiently and as soon as possible. This paper presents a review of the main concepts involving prevalence, etiology and classification of impacted canines, and describes the different clinical management options that would help solve the problem. It illustrates the topic by presenting a treatment delivered to a 13 year 7 months old male patient, suffering from a Class II, division 2, left subdivision, malocclusion, associated to a deep bite and a prolonged retention of a primary upper canine caused by the impaction of the permanent tooth. Corrective orthodontic therapy was associated to a rapid maxillary expansion and to the use of a high pull headgear. Impacted canine was submitted to orthodontic traction and correctly positioned back into the arch. This approach proved to be efficient in meeting both functional and aesthetic goals.

## RESUMO

O tracionamento de caninos impactados representa um dos grandes desafios da Ortodontia. Trata-se de um problema clínico relativamente frequente, cujo tratamento muitas vezes requer uma abordagem multidisciplinar. A exposição cirúrgica do canino impactado e a complexa mecânica ortodôntica aplicada para alinhar o dente na respectiva arcada podem, com frequência, levar a complicações nos tecidos de suporte, sem mencionar o longo tempo de tratamento e os custos elevados para o paciente. Por isso, vale a pena ressaltar a importância dos diagnósticos precoces, para que a interceptação do problema seja feita o mais cedo possível e de maneira eficaz. O presente trabalho apresenta uma revisão dos principais conceitos envolvendo a prevalência, etiologia e classificação dos caninos impactados, e descreve os vários tipos de abordagem clínica para a resolução do problema. Ao final, ilustra o tema com a apresentação de um tratamento realizado em um paciente de 13 anos e 7 meses de idade, portador de má oclusão de Classe II, divisão 2, subdivisão esquerda, associado a mordida profunda, que apresentava retenção prolongada de um canino superior decíduo, devido à impacção de seu sucessor permanente. O tratamento ortodôntico corretivo foi associado à expansão rápida de maxila e ao uso de aparelho extrabucal de tração cervical. O canino impactado foi tracionado ortodonticamente e posicionado corretamente na arcada. Essa abordagem foi eficiente para atingir as metas funcionais e estéticas propostas.

## INTRODUCTION

Among the main goals of an orthodontic treatment are enhancing facial and smile aesthetics, as well as improving masticatory function. In order to achieve these goals, it is definitely desirable, although not always possible, to have teeth being aligned according to a natural sequence.

Impacted upper canines often appears as a challenge against this goal for they play an important role in achieving good facial and smile aesthetics, given their strategic position over the canine eminences, which support both the alar base and upper lip. When properly aligned and with good shape and size, one gets very nice anterior teeth proportions and correct smile lines. Regarding the functional aspects, canines are equally important for supporting the overall dentition and for contributing to posterior disocclusion during lateral excursions.

Canines are the second most frequent cases of teeth displacement and impaction among all teeth, following third molar impactions.^1^ In general, they present an estimated prevalence that ranges from 1 to 4%.^2^ Impacted upper canines affect approximately 2% of the population and are twice as common in females as in males.^3^ The incidence of canine impaction is twice as high in the maxilla if compared to the mandible.^3^ Out of all patients who present impacted upper canines, 8% have bilateral impactions.^3^ In addition, two-thirds of all upper impacted canines affect the palatal aspect, while only one-third involve the buccal plate.

From a merely technical perspective, a given tooth is considered impacted when it remains infra-bony after its expected eruption period is due. When this position is off its normal eruption axis, as it often happens to canines, they are also considered displaced.^4^

There are two main theories associated to upper palatal canine impaction: the eruption guide theory and the genetic theory.^5^ According to the eruption guide theory, canines drill the eruptive movement along the lateral incisor root, that works as a guide through this eruption path. As a natural consequence to that, if the lateral incisors root is absent or malformed, canines might not erupt. The genetic theory, in turn, points out that genetic factors are the main cause of palatally displaced canine germs, including a couple of other possibilities associated to dental anomalies, such as lateral incisors agenesis and microdontia.^7^

Other authors would rather segregate the etiological factors for canine impactions into three groups: local, systemic and genetic factors,^8^ as shown in Table 1.

Among the local etiological factors, the ectopic position of the dental germ could be taken as the most important one; besides arch length discrepancies caused by lack of space; and the absence of an eruption guide, which is very common in cases of lateral incisor agenesis.^9^ However, the problem also seems to be associated with the long path that the canine germs have to overcome until its final eruption site.^3^

More specifically about space limitations, this seems to be the etiological factor more commonly related to labial upper canine impactions. Not by accident, one study showed that 85% of palatally impacted upper canines have enough space in the arch to erupt, while only 17% of labially impacted upper canines have enough eruption space.^9^

Early diagnosis and intervention during the mixed dentition phase can abbreviate treatment time, reduce costs and avoid more complex treatments to be performed during permanent dentition.^4^ The first diagnosis is undoubtedly the clinical one, where important signs may indicate the existence of the problem, namely: delayed eruption of the permanent canine; prolonged retention of deciduous canines after 14-15 years of age; absence of the labial canine eminence; presence of a palatal eminence; delayed eruption and distal tipping or abnormal migration of the lateral incisors.^3^ It is worth mentioning, however, that according to some authors, the absence of the canine eminence at earlier ages alone cannot be considered a predictive factor for canine impaction.^10^ In an evaluation of 505 students aged between 10 and 12, it was found that 29% did not present palpable canines at 10 years of age; only 5% at age 11; and at 12 years of age, 3% had the same condition. For this reason, in order to have an accurate diagnosis, the clinical examination should be complemented with imaging tests, such as x-rays and CT scans.^10^

The overall treatment time to move an impacted canine back to its place in the dental arch may vary considerably, depending on the complexity of the case. As a rule of thumb, one could expect approximately 12 months.^11^ Treatment duration is also related to the age of the patient. After puberty, it usually takes longer due to the greater mineral density of the bone tissue.^12^

**Table 1:** Canine impaction related etiological factors8.

Local factors
Dental arch discrepancies (lack of space)
Insufficient primary canine root exfoliation/resorption
Prolonged retention or early loss of primary canine
Ankylosed permanent canine
Cysts or neoplasms
Root dilacerations
Upper lateral incisor agenesis
Peg-shaped lateral incisor
Changes during lateral incisor root forming phase
Iatrogenic factors
Idiopathic factors
Systemic factors
Endocrine deficiencies
Feverish conditions
Irradiation
Genetic factors
Inheritability
Dental germ displacement
Palatal cleft affecting alveolar bone

Some sequelae may be caused by the presence of impacted canines: poor teeth positioning, either labially or palatally; migration of adjacent teeth into canine space, with subsequent loss in arch perimeter; internal resorption; dentigerous cysts; external root resorption of both impacted tooth and adjacent teeth; and infections accompanied by pain symptoms, mainly caused by partial eruptions.^13^

It is quite clear that the greatest risk is the possibility of root resorption in adjacent teeth. Studies using Cone Beam CT scan indicate that the percentage of root resorption in lateral incisors caused by impacted canines varies from 38%^14^ to 66.7%^15^.

Considering the usual absence of symptoms and clinician’s overconfidence of in 2D x-rays for dental impaction diagnosis, impacted canines associated with lateral incisors root resorption has a tendency to be later diagnosed, both in relation to the patient’s age as well as to the magnitude of the resorption. The limitations of 2D radiographic techniques are somewhat well known and include augmented images and other distortions, as well as structure overlaps. Approximately 37% of lateral incisors affected by root resorption appear normal on 2D^16^ radiographs. Therefore, CT scans represent the gold standard at present for the diagnosis of impacted teeth. It is possible to accurately identify and locate the impacted tooth position, to assess possible damage to the adjacent roots, and to quantify the bone around each tooth. The proper location of the impacted tooth plays a crucial role in determining the feasibility of good access to the surgical approach and the proper direction of orthodontic forces application.^4^

A higher risk of having adjacent teeth with root resorption caused by impacted canines is associated to cases where canines’ apexes remain open. This is justified by the fact that root development is directly related to the eruption process or dental migration. Once canine root development is complete, eruption or migration processes take place very slowly or even ceases, and canines become relatively steady. Consequently, impacted canines with fully formed root apexes are unlikely to be considered as a predisposing factor for the resorption of adjacent roots.

Another risk related to maintaining impacted canines is the possible formation of peri-coronal follicular cysts around its crown.^3^ In such cases, resorption of the neighboring roots may also occur if this enlarged follicle compresses blood vessels against the periodontal apparatus of adjacent teeth, causing cementoblasts to die along the affected root. When impacted canines are orthodontically displaced, the dental follicle usually moves off from adjacent teeth, which is usually sufficient to cease root resorption and trigger surface repair.

Some patients, however, even after properly warned by the professional and despite all risks concerning the maintenance of impacted teeth, may choose simply not to treat. In such cases, long term follow-up is recommended as to avoid having the impacted tooth causing pathological alterations. Patients should be informed that the long-term prognosis for the permanence of the deciduous canine is unfavourable, regardless of the aesthetic appearance of its clinical crown or the fact that it presents a good root length at the time of evaluation. In the vast majority of cases, roots undergo resorption over time and the deciduous tooth must be extracted.^4^

However, if the choice is for addressing the impacted canine problem, different approaches are described in the literature, namely: 1) extraction of the primary canine, allowing spontaneous eruption of the impacted permanent tooth; 2) extraction of impacted canine followed by implant placement on the site or orthodontic closure of the space; 3) orthodontic traction of impacted canine, with or without the need for previous surgical exposure; 4) autotransplantation of impacted canine. 

### 1) Extraction of the deciduous canine

Whenever early signs of a canine ectopic eruption are identified by orthodontists, an attempt should be made to prevent impaction and potential sequelae.^10^ Timely extraction of the primary canine is considered an effective measure in 80% of the cases in patients from 10 to 13 years of age, in particular if it is a palatal impaction.^17^

If the tip of the impacted canine cusp crosses the long axis of the lateral incisor, extracting the primary canine may not help in self-correction,^12^ or if it does at all, lower success rates, around 64%,^10^ should be expected. Now, if the tip of the canine cusp does not cross the long axis of the lateral incisor, there is a 91% chance of this canine erupting spontaneously after the removal of its predecessor.^10^ ^,^ ^18^

### 2) Impacted canine extraction

Impacted canine extraction followed by implant placement or orthodontic closure of the space is indicated for cases with poor prognosis: when the impaction is very deep, when canine root is completely formed, when a marked angulation (root laceration) is present, when there is too little space in the arch or when canine position is very unfavourable (between the lateral and central incisor roots, for instance), where the orthodontic movement of the teeth involved may harm others. In the above mentioned cases, extraction of the impacted tooth, replacing it by an implant or by the first premolar, can avoid considerable risks inherent to the orthodontic displacement, such as the difficulty in obtaining adequate bone and gingival levels, and the possibility of root resorption of adjacent teeth during this movement.^4^ ^,^ ^19^ Other indications for impacted canine extraction are: impacted canine cases that cannot be autotransplanted, internal or external resorption of the impacted tooth, acceptable and reasonably functional occlusion after first premolars replacement, or whenever a pathology is associated with the crown of the impacted tooth (cyst or infectious process).^4^

### 3) Orthodontic traction of impacted canine

The indication for the orthodontic traction of the impacted canine is more appropriate for cases with better prognosis, such as those of growing patients, without severe arch space deficiencies. Treatment involves the surgical exposure of the impacted tooth, followed or not by orthodontic traction, which will guide and align the tooth in the arch. Bone loss, root resorption and gingival recession around the pulled tooth are the most common complications of this type of procedure.^4^

In cases of surgical exposure aimed at triggering impacted canine displacement, good communication between the orthodontist and the surgeon is of the essence as to adopt the most appropriate technique. In order to choose the type of surgical exposure (open or closed) elements like impaction depth, anatomy of the edentulous area and the type of orthodontic force to be employed are some of the factors to be considered. The closed approach is strongly recommended as a treatment of choice when the tooth is impacted around the middle third of the alveolus or higher, in the vicinity of the anterior nasal spine. Since this approach replicates the natural tooth eruption, it is likely to provide the best aesthetic and periodontal results.^20^ In such cases, it is essential to remove as little bone tissue as possible when exposing the impacted tooth crown for bonding the traction accessory.^21^ In order to avoid having it placed at a very high gingival position, it is usually necessary to traction it from the palatal side, and special care should be taken regarding the direction of this traction.

The most common traction method for palatally impacted canines involves a surgical exposure followed by the bonding of the orthodontic attachment, so that a light and slow force can be applied to move the tooth along the correct position.^18^ For labially impacted canines, three surgical exposure methods are recommended, according to the position of the tooth in relation to the mucogingival junction: gingivectomy; apically rotated flap; and the closed surgical approach (in which a surgical exposure is made to access the crown and facilitate bonding, with immediate closure afterwards).^22^

Whenever using a bonded accessory and orthodontic force to pull the canine to its correct position, it is vital to keep in mind that first premolars should not be extracted until one is certain about the success in attempting to pull the impacted tooth; otherwise, the premolar is preserved and the canine is extracted.^4^

### 4) Autotransplantation of impacted canine

In selected cases, the orthodontist may also consider alternative treatment options like autotransplants, that should be performed under a multidisciplinary approach, gathering surgeons, periodontists, and prosthodontists. Patient should always be informed about the potential risks and complications inherent to the procedure.

## CASE REPORT

A 13 year 7 month-old male patient, during permanent dentition phase, presented with the chief complaint of a prolonged retention of the right upper primary canine without signs of mobility. He also complained about the “crooked smile,” where the right side was lower than the left side. Overall health signs showed no alterations. He reported a dental trauma in childhood, with the avulsion of the upper right primary incisor, followed by a nail-biting habit. No family history of dental impactions or severe dental ectopic events. Patient presented poor oral hygiene standards.

## DIAGNOSIS

**Figure 1:**
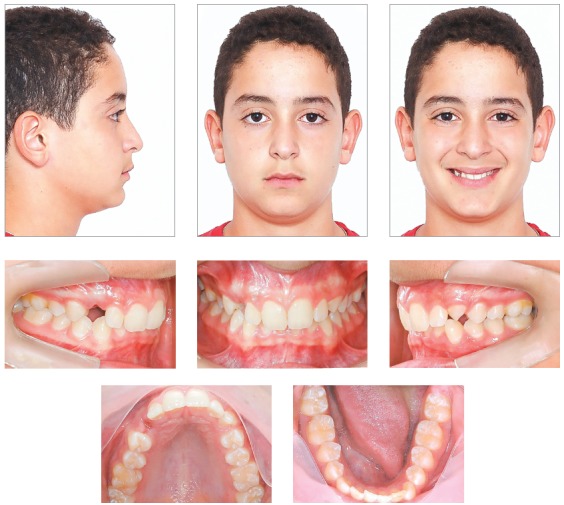
Initial facial and intraoral pictures.

Patient was seen to exhibit a convex facial profile, associated to a reduced lower third of the face due to a clear anteroposterior mandible deficiency. Despite an adequate nasolabial angle, the labiomental angle was lower than usual. Patient presented a good labial sealing at rest and there were no obvious asymmetries in frontal view. When smiling, he showed wide buccal corridors and little exposure of teeth (Fig. 1).

Anterior disocclusion was observed with incisal guide during protrusive movement and absence of canine dislocation guides during lateral movements, due to the poor position of upper canines. No premature contacts were observed.

An intraoral evaluation (Fig 1) revealed a left Class II, division 2 malocclusion, with upright incisors. Patient also presented increased overjet (3.0 mm) and very steep overbite, almost 100% (7.0 mm), with a tipped occlusal plane, lower on the right side. The upper right canine was absent in the arch and the upper left canine had erupted with a 45° rotation. No adequate space was observed for the correct alignment of the upper canines in the arch. A moderate crowding was also observed in the lower anterior region, as well as lower posterior teeth, that were lingually inclined. Upper midline was shifted about 1.0 mm to the left, and the lower midline was coinciding with facial midline. Masticatory muscles and temporomandibular joint were asymptomatic to palpation, with no clicking, crepitations or movement constraints.

**Figure 2:**
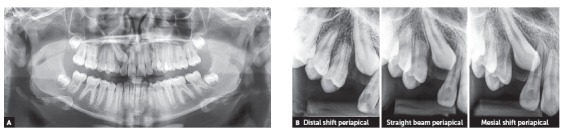
Initial radiographs: A) panoramic and B) right upper canine periapical radiographs (B).

Initial panoramic and periapical radiographs revealed the presence of all permanent teeth, with the exception of the impacted right upper canine and prolonged retention of the predecessor deciduous canine (Fig 2). Root contour, periodontal space and bony crests were shown to be normal.

**Figure 3:**
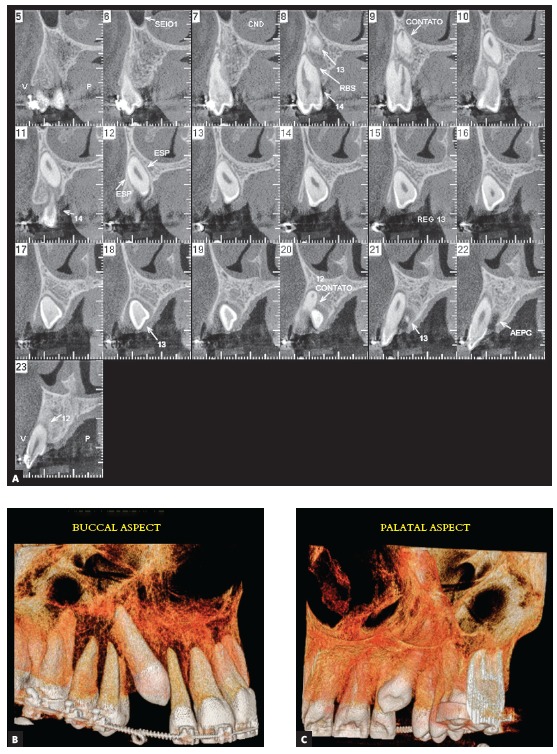
CT scan: (A) oblique cross-sectional slices and 3D reconstructions, buccal aspec (B) and palatal aspect (C).

The CT examination (Fig 3) confirmed the radiographic findings, where the right upper canine was mesially angulated, palatally displaced and in close contact with the neighboring teeth. Root resorption signs were already observed in the image of the first premolar.

**Figure 4:**
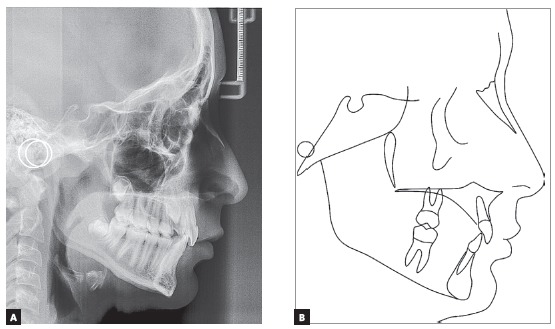
Initial profile cephalometric radiograph (A) and cephalometric tracing (B).

The cephalometric evaluation (Fig 4) and the measured values ​​(Table 1) revealed a Class II skeletal pattern (ANB = 6^o^, Wits = 6.0mm), due to a mandible set back (ANS = 78^o^, SNB = 72^o^, angle of convexity = 12^o^). The mandibular plane angle was displaced in the clockwise direction, as demonstrated by the SN.GoGn = 35^o^, which would only make it more difficult to correct the skeletal Class II. However, the Y axis was decreased (55^o^), what favoured the horizontal plane mandibular growth. The upper and lower incisors were vertically tipped (interincisal angle = 145^o^), with lower axial inclinations (1.NA = 14^o^, 1.NB = 17^o^, IMPA = 85^o^). 

## TREATMENT PLAN AND APPLIED MECHANICS

The planning of the case was carried out taking into account all facial, skeletal and dental aspects involved. For the upper arch, it was planned to start with the Haas palatal expander and perform activations once a day until the screw has reached 6.0 mm (about 24 activations). Then, after removal of the expander, full upper and lower fixed appliances were installed, with 0.022 x 0.028-in slot, including second molars. In the upper arch, teeth # 13 and # 23 were not included at first. Orthodontic bands were specifically designed for the first molars. Due to the deep overbite, posterior bite stops made with composite resin were used to lift the bite, allowing the bonding of the lower brackets. Concomitant to alignment and levelling, a high pull headgear was used for 10 to 12 hours a day, during night time, in order to promote distalization of the posterior teeth and to correct the skeletal Class II. Alignment and levelling was planned with the following sequence of archwires: coaxial 0.0175-in; NiTi 0.016-in; stainless steel 0.016-in; stainless steel 0.018-in; NiTi 0.019 x 0.025-in and stainless steel 0.019 x 0.025-in. In order to do space opening for the upper canines, nickel-titanium springs were planned between teeth #12 and #14 and between teeth #22 and #24. In order to correct molar asymmetry, the use of mechanics with 200g force (3/16-in heavy elastic) and Class II elastics was planned on a sliding jig, supported on tooth # 26. As soon as adequate space was obtained for the right upper canine, the patient was referred to do the surgical exposure and bonding of the orthodontic attachment (hook with metal chain). For the traction, a rectangular stainless steel 0,019 x 0,025-in archwire was planned, with a bypass in the region of tooth #13, with delta loop for the fixation of the elastic connected to the metal chain during traction. Once pulled, the hook was replaced by the orthodontic bracket on the buccal face of tooth #13, and NiTi 0.014-in and 0.016-in flexible archwires, overlapped with rectangular stainless steel archwires, were used to correct its position. The device should be repositioned during the finishing phase, guided by panoramic and periapical radiographs. Intermaxillary elastics were used to improve intercuspation. The following retainers were planned: removable wraparound appliance for the upper arch; fixed bar, bonded from canine to canine, with 0.0215-in coaxial wire, for the lower arch.

An alternative treatment plan could have been made if orthodontic traction of tooth #13 was not accomplished due to dental ankylosis or other factors. In this case, the solution would be the extraction of the impacted tooth and the opening of spaces in the region, for implant placement.

The prognosis of the case was considered positive considering the age of the patient, the favourable position of the impacted tooth and the periodontal biotype of the patient. 

## TREATMENT PROGRESSION

Treatment was performed exactly as planned, with an attempt to start it as soon as possible considering that the patient was almost 14 by then^23^ and that it would be necessary to open the medial palatal suture. But, as expected, the maxillary expansion went on smoothly.

The risk of failing to pull the impacted tooth due to dental ankylosis should never be overlooked.^24^ In addition, CT scan indicated an intimate relationship of tooth #13 with its neighbours, including signs of root resorption on tooth #14, which could hamper or prevent this movement. However, tooth #13 was surgically exposed without major problems and orthodontically pulled over a period of five months, with excellent patient collaboration in using the intermaxillary elastics and the high pull headgears appliance, what definitely contributed to treatment success.

The overall treatment time, including the rapid maxillary expansion, was 2 years and 4 months, with post treatment retainers applied as planned. Third molars were assessed at the end of the treatment and prescribed to be extracted. 

## RESULTS

**Figure 5:**
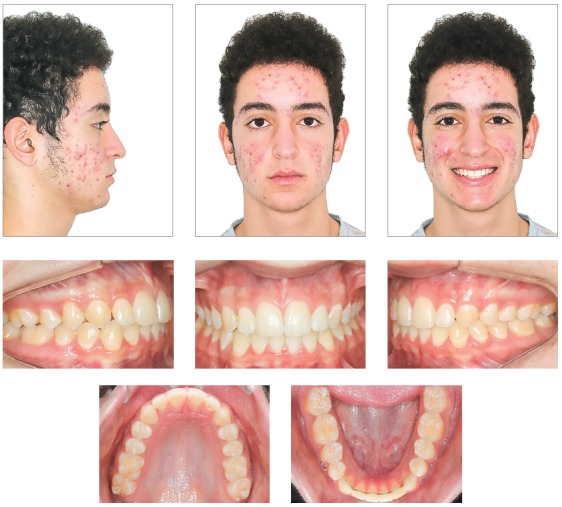
Final facial and intraoral pictures.

Considering the facial aspect, the profile improved considerably, with maintenance of the already satisfactory nasolabial angle, and improvement of the labiomental angle caused by mandibular growth. A better vertical dimension led to the correction of the deep bite that, in turn, caused the lower third of the face to be heightened and more vertically proportional.^25^ The smile became wider, but without the wide mouth corridors observed prior to the treatment.^26^ A better anterior teeth exposure led the smile to be expanded, which brought more cheerfulness to both the face and the smile altogether^27^ (Fig 5).

From the skeletal perspective, maxillary growth was restricted to the anteroposterior direction while some mandibular growth occurred, leading to a significant improvement in the relation between the bony bases in the sagittal direction.^25^ ^,^ ^26^ In vertical terms, neither posterior mandibular growth or rotation were observed, what could have further increased the mandibular plane angle. Quite the opposite was observed, with a reduction of the mandibular plane angle, and a significant improvement in facial profile^25^ (Fig 8). In summary, the cephalometric superposition revealed that a good deal of facial growth was experienced, with the entire face moving forward and downward (Fig. 9).

**Figure 6:**
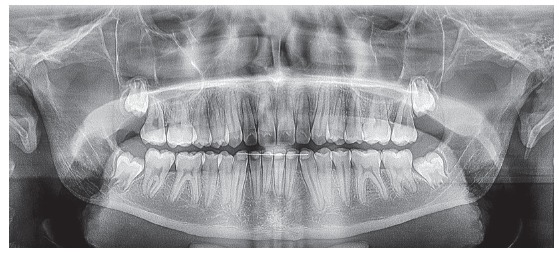
Final panoramic radiograph.

**Figure 7:**
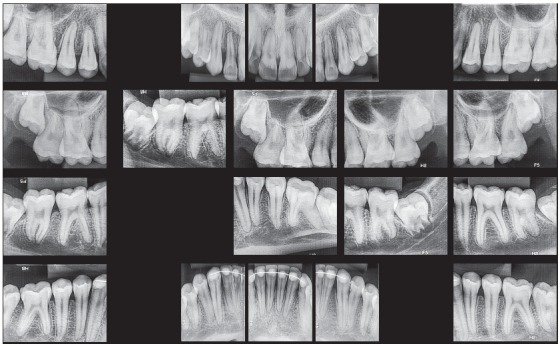
Final periapical radiographs.

As for teeth positioning and occlusion, a significant improvement was seen in anterior teeth position in their bony bases, obtained by the expansion of both arches and the application of ideal torques. The improvement in 1-NA and 1-NB cephalometric measures ratifies this alteration (Table 1). A better levelled curve of Spee caused lower incisors to protrude (Fig 9). It is well known that for every 1.0 mm of change in the curve, a 4^o^ increase in the inclination of the inferior incisors should be expected.^28^ No periodontal apparatus injuries were observed, even after relatively large labial movements. Dental intercuspation was improved, especially on the left side, that started out with a Class II malocclusion. The sliding jigs mechanics and Class II elastics proved to be efficient in correcting the dental asymmetry.^29^ The Class I relationship between the inclined planes was rather satisfactory as well. Overbite and overjet were corrected together with the midline deviation. Dental element #13 was successfully tractioned, causing no harm to the neighbouring teeth (Figs 6 and 7).

**Figure 8:**
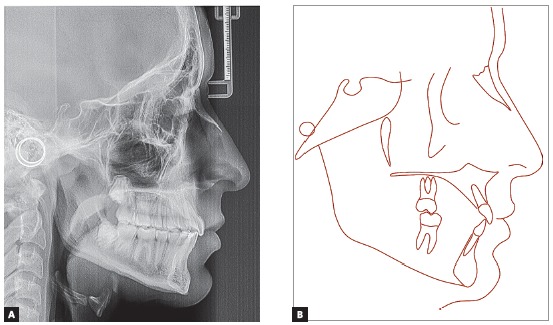
Final profile cephalometric radiograph (A) and cephalometric tracing (B).

**Figure 9:**
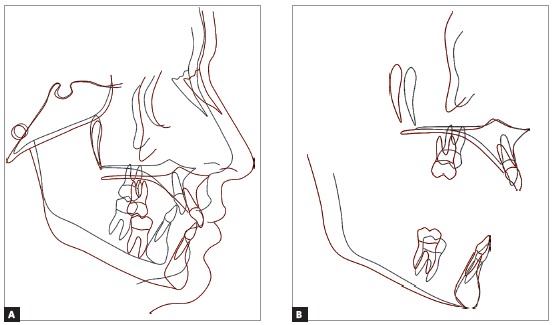
Total superposition (A) and partial superposition (B) of the initial (black) and final (red) cephalometric tracings.

**Table 1:** Initial (A) and final (B) cephalometric values.

	Measurements		Normal	A	B	A/B dif.
Skeletal pattern	SNA	(Steiner)	82°	78°	78°	0
	SNB	(Steiner)	80°	72°	77°	5
	ANB	(Steiner)	2°	6°	1°	5
	Wits	(Jacobson)	♀ 0 ±2 mm ♂ 1 ±2 mm	6 mm	2 mm	4
	Angle of convexity	(Downs)	0°	+12°	0°	12
	Y-axis	(Downs)	59°	55°	55°	0
	Facial angle	(Downs)	87°	88°	92°	4
	SN-GoGn	(Steiner)	32°	35°	31°	4
	FMA	(Tweed)	25°	24°	22°	2
Dental pattern	IMPA	(Tweed)	90°	85°	100°	15
	1.NA (degrees)	(Steiner)	22°	14°	28°	14
	1-NA (mm)	(Steiner)	4 mm	1 mm	5 mm	4
	1.NB (degrees)	(Steiner)	25°	17°	28°	11
	1-NB (mm)	(Steiner)	4 mm	3 mm	5 mm	2
	$\begin{array}{c} 1\\ 1 \end{array}$ - Interincisal angle	(Downs)	130°	145°	123°	22
	1-APo	(Ricketts)	1 mm	3 mm	3 mm	0
Profile	Upper lip - S-line	(Steiner)	0 mm	0 mm	-1 mm	1
	Lower lip - S-line	(Steiner)	0 mm	0 mm	0 mm	0

Last but not least, regarding the functional aspect, some improvement in the masticatory function was observed, alongside with good gingival and periodontal health, that presented adequate gingival contours. All things considered, the stomatognathic system health in general was preserved. Simultaneous bilateral equipotent alternating contacts in centric relation and in maximum intercuspation were accomplished, together with normal disocclusion parameters during mandible excursion movements. No occlusal adjustments or selective wear were needed to refine the occlusal contact distribution.

Panoramic, periapical and interproximal radiographs revealed no cavities or endodontic problems, roots with preserved contours and good parallelism, with alveolar bone crest heights preserved were observed (Figs 6 and 7). The final result was quite satisfactory, and the treatment was rigorously delivered within the planned time: 4 months for rapid maxillary expansion and 24 months for fixed corrective orthodontics. 

## DISCUSSION

Orthodontic management of impacted canine may offer considerable challenges. Therefore, good tomographic images are fundamental to a successful traction, for they allow professionals to accurately identify and locate the position of the impacted tooth, evaluate possible injuries to adjacent roots and to quantify the bone around each tooth. It also helps in detecting the existence of possible ankylosis in the roots of such teeth,^24^ which could be interpreted as the most probable cause for them failing to reach the expected position during the eruptive movement. The visualization of these ankylosed zones may help professionals to choose a different treatment protocol, if compared to the conventional surgical exposure followed by orthodontic traction. This will assist in the right choice for either the impacted tooth extraction, autotransplantation or the execution of a deep alveolar corticotomy followed by immobilization, especially if the canine is more labially positioned.^30^

In the clinical case presented, the post treatment image evaluation did not show signs of external root resorption of neither the tractioned tooth nor adjacent teeth, despite some studies showing this to be the main side effect related to orthodontic traction of impacted teeth.^31,32^ One of the possible explanations for the absence of these problems may be the lack of extensive movements of the impacted tooth, with ideal pulling forces being applied during an adequate treatment period.^33^. Once again, no doubt remains about the benefits of using CT scans, which improved predictability and accuracy^32^ for diagnostic purposes.

Regarding the choice for the surgical exposure method, the correct diagnosis led to the conclusion that the closed approach was the most appropriate one. Some authors recommend this approach because it may possibly spare patients from a new periodontal surgical procedure, with less tissue manipulation of the dental follicle, an important structure for eruption process. It also allows traction forces to be applied on the long axis of the tooth.^33^ The canine traction towards the palatal direction avoids buccal alveolar bone loss and prevents contact with the roots of adjacent teeth, as recommended by the literature.^34^ Palatally displaced canines, when submitted to orthodontic traction, involve less periodontal apparatus if compared to labially displaced canines.^35^ The palatal approach avoids injuries to the buccally attached gingiva, preventing gingival recession, decreasing the chances of bacterial plaque accumulation.^36^

## CONCLUSION

An adequate management of impacted canines, from both functional and aesthetic perspectives, is of utmost importance for the overall success of the orthodontic therapy. Dental professionals must select, among the many available treatment plan options, the one that best meets patient’s needs and interests. The most suitable method to be chosen by the orthodontist should be one that allows the application of ideal traction forces in the most favorable direction, avoiding further injuries to adjacent teeth.
